# Single Virus Genomics: A New Tool for Virus Discovery

**DOI:** 10.1371/journal.pone.0017722

**Published:** 2011-03-23

**Authors:** Lisa Zeigler Allen, Thomas Ishoey, Mark A. Novotny, Jeffrey S. McLean, Roger S. Lasken, Shannon J. Williamson

**Affiliations:** 1 Microbial and Environmental Genomics, J. Craig Venter Institute, San Diego, California, United States of America; 2 Scripps Institution of Oceanography, University of California San Diego, La Jolla, California, United States of America; Institut Pasteur, France

## Abstract

Whole genome amplification and sequencing of single microbial cells has significantly influenced genomics and microbial ecology by facilitating direct recovery of reference genome data. However, viral genomics continues to suffer due to difficulties related to the isolation and characterization of uncultivated viruses. We report here on a new approach called ‘Single Virus Genomics’, which enabled the isolation and complete genome sequencing of the first single virus particle. A mixed assemblage comprised of two known viruses; *E. coli* bacteriophages lambda and T4, were sorted using flow cytometric methods and subsequently immobilized in an agarose matrix. Genome amplification was then achieved *in situ* via multiple displacement amplification (MDA). The complete lambda phage genome was recovered with an average depth of coverage of approximately 437X. The isolation and genome sequencing of uncultivated viruses using Single Virus Genomics approaches will enable researchers to address questions about viral diversity, evolution, adaptation and ecology that were previously unattainable.

## Introduction

Whole genome amplification and sequencing of single microbial cells is a powerful new tool in the field of microbial genomics, enabling direct examination of the genomic contents of individual cells without the need of cultivation [1]–[3]. Microbes are found in nearly all environments (e.g., human microbiome, rhizosphere, aquatic ecosystems, soils, air) performing essential roles in processes such as biogeochemical cycling [4], degradation [5], metabolism [6], and forming the foundation of environmental food webs [7]. Sequencing of single cells permits the study of previously uncharacterized microbes from virtually any environment, thus enabling the direct and comprehensive analysis of a microbe's genetic and metabolic repertoire. Flow cytometry [1], [8] and micromanipulation [9], [10] have aided in the advent of single cell isolation and sequencing by providing access to individuals from naturally occurring consortia or pure cultures. A reaction called multiple displacement amplification (MDA) [11]–[13], which uses the high-fidelity processive capabilities of the phi29 DNA polymerase, can amplify the genome of a bacterial cell more than a billion-fold generating the micrograms of genomic DNA typically required for DNA sequencing either via Sanger sequencing [14], 454 pyrosequencing [15], and/or Illumina platforms [16]. While some sequence is lost due to non-specific amplification or damage to the single genome copy, as much as >90% of the genome has been recovered from single cell sequencing [16], [17]. Small MDA reaction volumes were shown to improve amplification from single viral DNA molecules [18] and single cells [15]. Recently, Rodrigue *et al*., showed a consistent increase in the total genome coverage of *Prochlorococcus* single-celled amplified genomes by using a duplex-specific nuclease to degrade highly abundant sequences apparent after amplification; thereby increasing the coverage of low abundant sequences.

**Figure 1:**
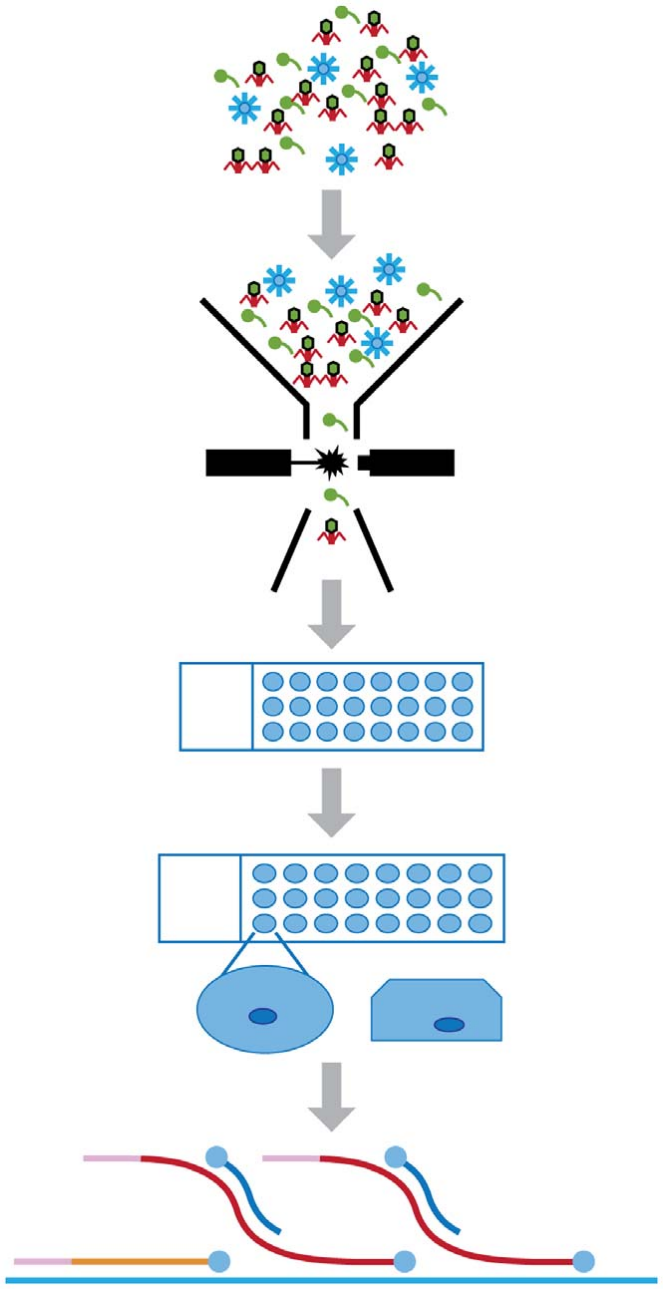
Flow diagram depicting SVG methodology. Viral suspensions are sorted via flow cytometry onto PTFE slides with 24 distinct wells containing agarose beads. Viral particles are then embedded within the agarose bead by overlaying with an additional layer of agarose. Lastly, MDA is performed *in situ*.

While most single cell studies have focused on bacteria and cyanobacteria, single virions have yet to be isolated and genomically described using similar mechanisms. Viruses are ubiquitous and the most numerous and diverse biological entities on our planet [19]. Nearly all aspects of our lives are influenced by viruses through shaping the environments that surround us [20], our immune responses [21] and even our genomes [22]. The field of environmental viral metagenomics has gained momentum over the past several years [23]–[28]; however, sequencing of individual environmental viral genomes is currently dependent on the establishment of cultivable virus-host systems. With this in mind, if less than one percent of microbial populations can be cultured using standard microbiological techniques due to incongruencies in direct counts versus cultivatable microbes [29]–[32], then only a very small number of viruses have the likelihood of being genomically described. Currently, viral genomic sequences are lacking in public databases, with the exception of human viruses and those of agricultural and industrial significance (e.g. Lactococcal phages). Clearly, a better understanding of virus diversity and evolution will not be achieved until the genomes of a broad range of viruses are available. Here we introduce an approach for isolating and characterizing the genomes of viruses called “Single Virus Genomics” (SVG) (Figure 1). The benefits of SVG will be far-reaching, enabling novel virus discovery in a variety of clinical and environmental settings, altering our understanding of virus evolution, adaptation and ecology and facilitating the interpretation of viral genomic and metagenomic data by providing suitable reference genomes.

## Results

### Single virus isolation

**Figure 2:**
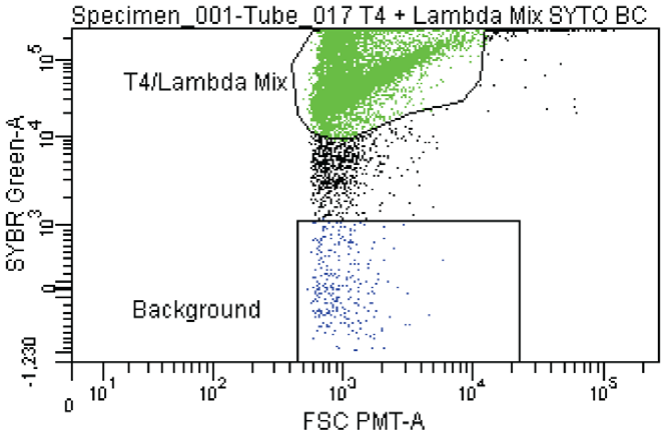
Flow cytometric bi-plot showing SYBR-stained T4 and lambda phage mixture. Gates were chosen to highlight T4/lambda assemblages (green), and background (blue). Particles not gated (black) were not sorted.

Flow cytometric methods have been optimized for [33] and used on natural viral populations for enumeration purposes [34]–[36]. Therefore, for this study, flow cytometry was used to sort a mixed viral assemblage consisting of two known viruses; *E. coli* bacteriophages lambda and T4. To increase the accuracy of detecting a single viral particle, a fluorescence-activated cell sorter (FACS) AriaII with a forward scatter photo multiplier tube (PMT) was used, which enabled more sensitive detection and lower size thresholds (Figure 2). While 96- and 384-well microtiter plates would have been optimal for high-throughput processing of viral assemblages, we were unable to reliably recover single virions from plate wells. The majority of wells (98%) contained no viruses evidenced via polymerase chain reaction (PCR) amplification of specific loci for each bacteriophage. Therefore, as an alternative strategy, viruses stained with SYBR Green (Invitrogen) were sorted directly onto cooled agarose beads applied to ‘multi-well’ polytetrafluoroethylene (PTFE) microscope slides to increase virus capture efficiency, as well as, to maximize the recovery of high-quality template DNA required for the MDA reaction. PTFE slides were chosen due to the ability of defining each sorting event since they possess distinct regions (wells) where agarose beads were positioned. Overlaying the nanoliter droplet containing the virions with additional agarose simultaneously embedded and stabilized the sorted viral particles in preparation of downstream processing.

### Single virus validation: Confocal microscopy and loci-specific PCR

**Figure 3:**
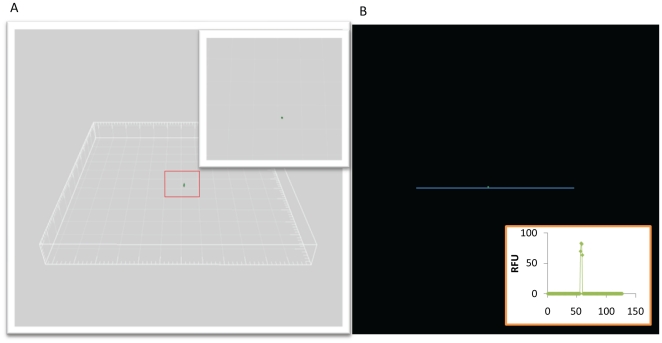
Confocal laser scanning micrograph of sorted viral particle embedded in agarose bead. A) Three dimensional reconstruction of syber green I stained viral particle within depth of agarose bead verifying a single sorted event. Inset: higher magnification of viral particle. B) Profile plot of relative fluorescence for a stained viral particle in an agarose bead. The blue line through the viral particle (green) is the reference for the inset graph showing the relative fluorescence.

**Figure 4:**
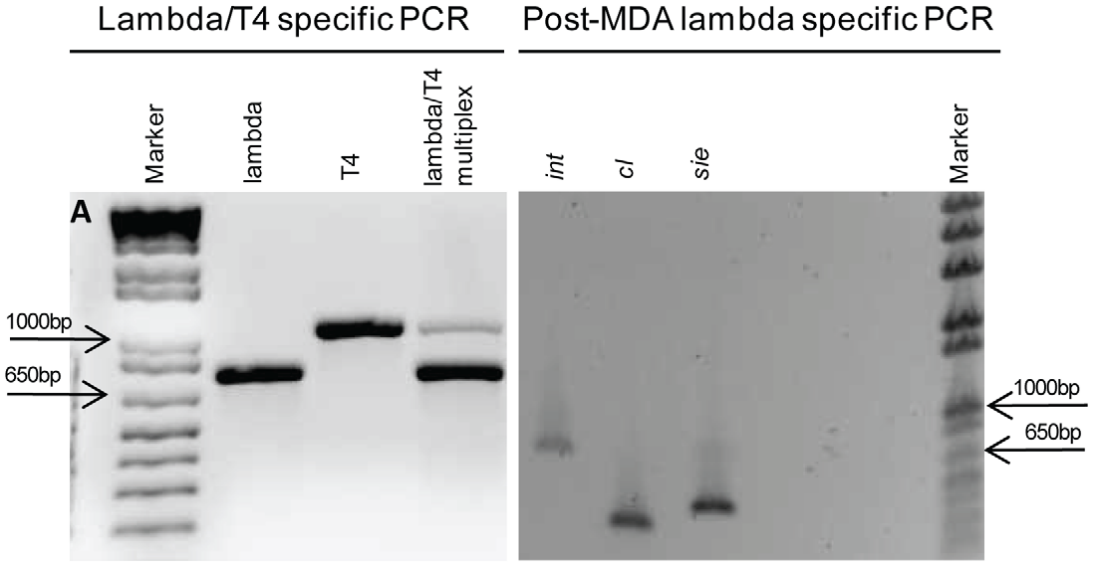
Phage identification using PCR. A) Multiplex PCR using T4 and lambda-specific primers to genotype, Lanes: 1. TrackIt 1 kb plus ladder (Invitrogen), 2. Lambda integrase (750 bp), 3. T4 major capsid protein (1050 bp), 4. Mix of lambda integrase and T4 major capsid protein. B) Subsequent lambda specific PCR with additional loci to further confirm phage genome isolation, Lanes: 1. Lambda integrase (750 bp), 2. Lambda repressor (356 bp) 3. Lambda sie (superinfection exclusion) (456 bp) 4. TrackIt 1 kb plus ladder (Invitrogen).

To confirm isolation of single viruses, Confocal Laser Scanning Microscopy (CLSM) was performed to detect the fluorescently stained virions embedded in agarose [37], [38]. CLSM was chosen to obtain greater confidence that only a single viral particle was contained within an agarose bead through 3D rendering of stacked images surrounding the viral particle (Figure 3A). Additionally, Figure 3B demonstrates the utility of CLSM to detect the relative fluorescence of a single stained virus particle above background. Once successful candidates were identified, whole genome amplification via MDA was performed *in situ* in order to minimize the potential for virus loss, reduce genomic shearing, and contamination. Multiplex PCR using T4 (gp23, major capsid gene) and lambda (integrase gene) specific primers was performed on the amplified genomic material to confirm the genotype of the virus and indicated that the isolated viral particle used in this study was phage lambda (Figure 4A). To further confirm this result, two additional loci specific to lambda were targeted including the superinfection exclusion protein B and repressor genes (Figure 4B). The results confirmed our initial finding that we had isolated and amplified the genome of phage lambda.

A subsequent experiment to quantify virus particles within agarose droplets using CLSM indicated that 75% contained 1 or >1 (1–5), viruses (Table S2); and amplification of genomic material via MDA was successful in 92% of virus-containing droplets. Multiplex PCR using T4 and lambda-specific primers on amplified genomic material was successful for 25% of the droplets and positive results were only found for those droplets containing one or more virus.

### Sequencing, reference mapping and *De novo* assembly

**Figure 5:**
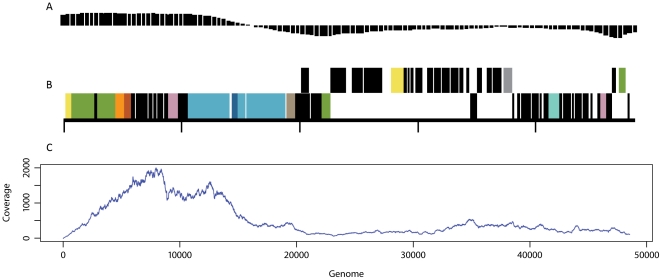
Lambda genome attributes and coverage. A) GC plot with bars indicating %GC above or below the average of 49%, B) Genome map of lambda (adapted from http://img.jgi.doe.gov), and C) Reference mapping of SVG reads to phage lambda, x-axis is genome position, y-axis is %coverage.

The 48,502 bp double stranded DNA phage lambda was sequenced using 454 GS FLX titanium pyrosequencing to an average coverage of 437X across the genome (Figure 5C), (ranging from 0–2000X). With the exception of the first 5 bp, the complete genome of lambda was recovered (Table S3). Lacking the first 5 bp is likely due to a reported artifact of MDA reactions where the ends of linear DNA segments are underrepresented [12], [39]. It has been reported that MDA is biased against genomic areas of high GC content [40], however, our data suggests otherwise as there was higher coverage in the regions of greater %GC, shown in Figure 5A where the bars indicate the GC above or below the average (average GC of phage lambda is 49%). We expected to achieve >600X coverage from the 99,911 sequencing reads (mean read length of 361.6 bp) that were generated if all sequences produced were from the lambda template DNA. Reference mapping to the lambda genome (NCBI Accession J02459) indicated that 38,505 (38.5%) reads did not recruit to the genome and were further termed ‘unmapped’. BLASTX analysis was performed on these sequences, which resulted in 22,411 reads (58.2% of the unmapped set) annotated based on homology to public sequences (Table S4). The stringent settings used during reference mapping prevented the recruitment of 116 sequences classified as *E. coli* lambda phage through BLAST. The majority of the annotated unmapped sequences were classified within the *Pseudomonas* genera (12.9% of total reads). To assess errors within the amplified lambda genome SNP analysis was completed, which resulted in the detection of 17 SNPs across the genome (Table S6), however, it is difficult to determine if these errors arose during amplification, 454 pyrosequencing, or maintenance of ATCC cultures. Deletion-Insertion Polymorphisms (DIPs) are also given in Table S5; Two DIPs corresponding to reference positions 31619 and 39143 are deletions causing a frameshift within essential phage proteins. The deletions (-) are found in 37.5 and 38.6 percent of the reads covering the corresponding positions in the lambda repressor (cl) and DNA replication proteins respectively. It is likely, therefore, that the polymorphism would not be present in the phage population but perhaps are a result of MDA and/or 454 pyrosequencing artifacts.

**Figure 6:**
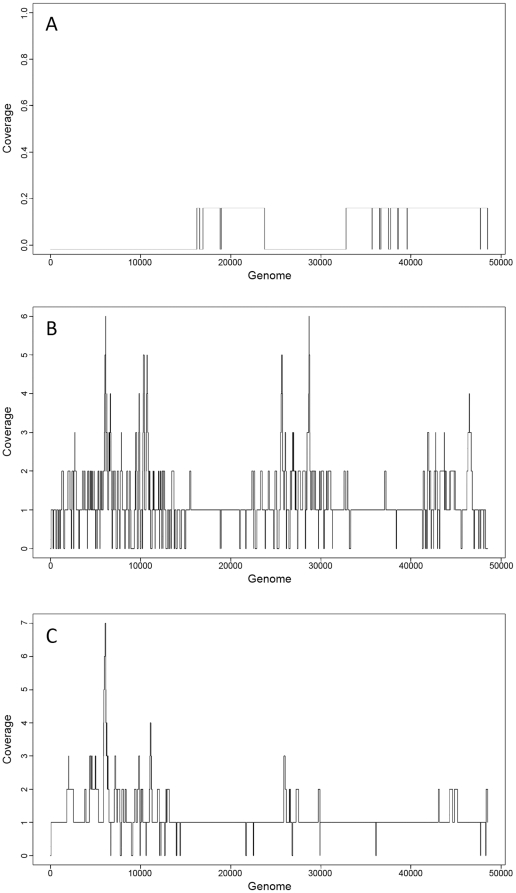
*De novo* assembly of reads followed by reference mapping to evaluate assembly. A) Filtered sequences randomly to 3400 reads, approximately 22X coverage of the lambda genome, B) All reads (99,911), C) Normalization of assembly by reducing redundancy of overrepresented sequences from (B).

*De novo* assembly was performed with the GS De Novo Assembler Software (i.e., Newbler, 454 Life Sciences) to assess the utility of these methods for use on unknown SVGs (Table S6). Optimal coverage depth for assembly is between 15–25X (personal communication 454 Life Sciences; Newbler manual); therefore the number of reads was randomly reduced to yield 30X (4,700 reads) and 22X (3,400 reads) coverage, with the latter generating the highest quality assembly (Figure 6A). Although reducing coverage resulted in the highest quality assembly based on assembly metrics (i.e., fewer contigs, longer length, greater N50 [41]-see methods for details), using all reads (Figure 6B) resulted in near complete coverage of the genome, however with shorter contigs. In an effort to increase contig size while retaining genome coverage, the redundancy of reads among overrepresented contigs from this assembly was reduced and the data re-assembled, as seen in Figure 6C [16]. This method of bioinformatic normalization of the data resulted in larger contigs coupled with almost complete coverage of the genome (>99%). The utility of SVG approaches for the study of uncultivated viruses will ultimately depend on the success of *de novo* assembly due to the lack of suitable reference genomes. Recently, SVG approaches were applied to virioplankton samples collected from the Southern California Current (Zeigler Allen, et al., in prep) followed by bioinformatic normalization during *de novo* assembly procedures, which similarly improved assembly statistics.

## Discussion

The Single Virus Genomics approach described here enabled, for the first time, isolation and whole genome sequencing of an individual virus; a significant technical achievement that has the potential to alter the course of virological research. Further optimization of SVG will pave the road to high throughput processing of uncultivated viral assemblages, advancing studies of viral diversity, evolution, adaptation and ecology. These include efforts to improve the occurrence of single virus particles in agarose droplets. Although genotyping of the single lambda phage particle that yielded the sequence data for this study was successful (Figure 2) and can clearly be accomplished, the overall low success rate (25%) of specific PCR post-MDA evidenced was possibly due to a lack of purification of the MDA products prior to genotyping with T4 and lambda-specific primers, which is recommended (see materials and methods). In addition, the high success rate of MDA (92%), as evidenced by gel electrophoresis, could represent non-specific amplification in addition to amplified viral DNA. Optimization of flow-sorting parameters should increase the likelihood of capturing individual viruses.

While complete coverage of the lambda phage genome was our goal, the first 5 bp of the genome were missing, perhaps due to DNA breakage or the linear nature of the molecule. To date, complete coverage of bacterial genomes has not been reported, suggesting that single cell genomics projects suffer from similar obstacles. We make an effort to reduce DNA breakage through the immobilization of viral particles in an agarose matrix which minimizes DNA damage during viral particle isolation and genome amplification. When applying SVG techniques to unknown viruses, it may be difficult to determine if the ends of linear genomes have been captured. However, approaches for genome closure such as primer walking could be attempted on the amplified viral genomic material if complete coverage is critical. A recent study of MDA on phage lambda genomic DNA also showed underrepresentation of DNA termini and reported using a ligation reaction prior to MDA to generate circular molecules, thereby overcoming this bias[42]. A similar approach can be adopted if future data suggests it is necessary.

Background DNA synthesis or nonspecific amplification is commonly reported during amplification using the MDA reaction [18]. Nonspecific amplification has been attributed to contaminating DNA emerging from reaction reagents and/or through a mechanism that enables amplification from the random hexamers within the reaction mixture. The average coverage retrieved here was lower than expected, most likely due to non-specific amplification. As mentioned previously, steps were taken to reduce the likelihood of contaminating DNAs being introduced into our sample following flow cytometric sorting. However, during the sorting process we acknowledge that free DNA as well as multiple viral particles may be co-transported. Treatment of viral assemblages with DNase I and examination of virus containing agarose beads using confocal microscopy was used to address these issues. Additionally, there is a higher likelihood of nonspecific DNAs preferentially amplified due to the lower quantity of template viral DNA as opposed to single bacterial cells as a result of the significant difference in particle (cell) size and genomic DNA content (25–100 nm; ∼1.5femtograms for viruses, as opposed to 0.2–1.5 um; ∼14femtograms for bacteria). To address this potential shortcoming the incubation time of genome amplification was reduced and we took advantage of the massively parallel, high-throughput capabilities of pyrosequencing to ensure both adequate coverage of the lambda genome and to examine the nature of any nonspecific amplification. A potential source of contaminants is high molecular weight DNA fragments present in commercially available phi29 polymerases. A recent report found no manufactured enzyme to be contaminant-free and that levels of contamination varied among enzyme and buffer reaction lots [43]. Specific 16S bacterial DNA sequences were detected as contaminants in our process. The identity of the microbial contaminants present in no template control (NTC) MDA reactions using 16s rDNA PCR and sequencing were determined and the most abundant taxa are similar to those found in our taxonomic classification of unmapped reads, in particular *Burkholdaria* and *Pseudomonas* (Table S7). We have not attempted to distinguish between the particle handling, MDA, 16S PCR and PCR product sequencing steps as the potential source of the contaminants. While bioinformatic curation of data can be performed to identify potential contaminants that are not related to the target viral molecule, conservative approaches are necessary in their removal so that pertinent data is not lost. Therefore, steps to reduce the amount of exogenous DNA-based contamination prior to sequencing are imperative and are especially relevant when working with unknown viral isolates. For example, testing new enzyme and reagent lots prior to use and the reduction of free DNA through nuclease treatment should help to reduce nonspecific amplification.

A number of important factors must be taken into consideration when applying SVG approaches to natural, unknown assemblages of viruses. Although it is possible to capture and immobilize RNA-containing viruses using flow cytometry, the MDA reaction will not work on RNA templates. However, a reverse-transcription step prior to amplification would address this issue and we are currently evaluating the utility of whole transcriptome amplification (WTA) to amplify individual RNA viral genomes. Genotyping of previously unknown viruses is another topic that deserves careful consideration since PCR using virus-specific primers conserved across all viral groups is not option. While certain techniques such as randomly amplified polymorphic (RAPD) PCR may successfully produce unique viral genomic fingerprints [44], we are also evaluating alternative strategies such as optical mapping [45] and automated artificial neural networks using known morphological characteristics and fluorescence data gathered from reference phage for training [46].

Single virus genomics has the potential to dramatically influence a wide variety of fields that will benefit from whole genome sequence data produced from previously uncultivated viruses; including (but not limited to) viral and microbial ecology, evolutionary biology, epidemiology, immunology and other clinical and agricultural-based sciences. In addition to enabling novel virus discovery and facilitating comparative genomic analyses, SVG will also provide an ‘anchor’ for metagenomic studies by supplying relevant reference genomes. Reference viral genomes will not only assist assembly of metagenomic data, but will help to address questions surrounding genetic and functional biodiversity, as well as the representation of individual viruses within a community. Lastly we anticipate that the production of new reference viral genomes will improve our ability to classify previously unidentified sequences within viral metagenomes, effectively bridging the gap between genomic and metagenomic studies.

## Materials and Methods

### Viral suspensions

Bacteriophage standards for T4 and lambda were obtained from ATCC (ATCC 11303-B4 and 23724-B2, respectively). Stocks were diluted in 0.1 µm-filtered TE (Tris-EDTA, pH 7.2, Invitrogen) followed by filtration through a 0.22 µm Pall syringe filter. The viral particles were not fixed prior to flow cytometry, as is typical, due to insufficient evidence that the fixative would not inhibit downstream reactions.

### Flow cytometry parameters

Viral particle suspensions were sorted on a BD FACSAria II Flow Cytometer equipped with a custom Forward Scatter PMT (FSC PMT). The particles were diluted in 0.1 µm filtered TE (Tris-EDTA, pH 7.2, Invitrogen) to an appropriate titer for an event rate of 200 events s^−1^. TE was used because it improves the emission signal of stained viruses [33]. Thresholds were set to FSC PMT at 1000 and SSC at 200 for T4/lambda particles to maximize signal-to-noise ratios. Prior to beginning the sorting, blanks containing 0.1 µm-filtered TE were measured for background event recognition. In addition to blanks, unstained and stained viral particles of the sample were measured to a total of 5,000 events each. Readings were measured on bi-exponential plots, consisting of a lower linear scale and a higher exponential scale. Viral particle suspensions were stained with SYBR Green I (Invitrogen) and sorted onto polytetrafluoroethylene (PTFE) printed microscope slides (Electron Microscopy Sciences). These slides were chosen due to their hydrophobic feature, which controls for cross-contamination and low microliter capacity. Data was analyzed using the BD FACSDiva Software v.6.1 software package.

### Agarose immobilization

Twenty-four well PTFE slides were used for agarose immobilization. To each well, 5 µl of low melting point (LMP) agarose, cooled to 37°C, was added. The viral particle suspensions were subsequently sorted directly onto the LMP agarose droplets at a concentration of 1 event per well. Each well was then overlaid with 5 µl of LMP agarose, cooled to 37°C, thus embedding the virions.

### Visualization and whole genome amplification

The embedded virion(s) were stained with SYBR Green I and visualized on the slide using Confocal Laser Scanning Microscopy (CLSM) to determine that a single viral particle was present in each agarose droplet. CLSM was performed with a Leica TCSP5 (Leica Microsystems) with 488 nm laser excitation. Image stacks were obtained using a 63× long working distance objective, which enabled visualization of the viral particle in the depth of the agarose plug. Simulated 3-D images and sections were generated using the software Volocity and the plan views with side profile slices using IMARIS (Bitplane AG, Zűrich, CH). Once a well was identified as positive, the single viral particle was lysed using heat (94°C) for 3 minutes and its genomic material amplified *in situ* using the phi29 DNA polymerase and multiple displacement amplification (MDA) reaction, as per manufacturers recommendations (GenomiPhi kit, GE Healthcare). After amplification, the genomic material was purified away from the agarose matrix using a β-agarase (New England Biolabs) reaction followed by purification using buffer-saturated phenol (Invitrogen) and ethanol precipitation. Unincorporated dNTPs and random hexamer primers were removed through column purification according to manufacturer specifications, (PureLink Genomic DNA Purification, Invitrogen) as they would be a source of contamination on downstream reactions, such as sequencing. We highly recommend the previous step as it was needed to reliably obtain successful specific PCR results (see below). An additional round of MDA, restricted to one hour, was performed in triplicate, pooled and the amplified genomic DNA purified as described above.

### Multiplex PCR

Multiplex PCR was used for validation of model bacteriophage isolation and genotyping. Primer sets specific to the lambda integrase and T4 bacteriophage gp23 major capsid genes were mixed and used in gradient PCR to identify the annealing temperature for subsequent reactions (Table S1). PCR was performed using Platinum® Taq DNA polymerase, HiFi (Invitrogen). Additional genes were PCR amplified to verify the lambda genotype, which included the lambda repressor (rep) and superinfection exclusion (sie).

### Library construction and sequencing

Purified amplified genomic DNA was randomly sheared using nebulization and ends polished using BAL31 nuclease (New England Biolabs) and T4 DNA polymerase (New England Biolabs) reactions. Fragmented DNA was size selected using gel electrophoresis and 1% low melting point agarose. The DNA was purified from the gel using β-agarase (New England Biolabs) followed by buffer-saturated phenol extraction and ethanol precipitation. Libraries for 454 pyrosequencing were constructed using the sheared DNA. AMPure size fractionation was used to purify the above reactions, followed by ligation of 454 adaptors and emulsion PCR (ePCR). Sequencing was performed using the 454-Titanium protocol.

The Nucleotide sequences were deposited as raw reads in GenBank under the accession number SRA029358.

### Reference mapping and *De novo* assembly

Reference mapping was conducted using CLC Genomics Workbench, using the Enterobacteria phage lambda (ACC J02459.1). Assembly parameters were as follows: local alignment with mismatch cost 2, insertion cost 3, deletion cost 3, length fraction 0.5, and similarity 0.9. Therefore, 50% of the read needed to be aligned at 90% similarity. Parameters for SNP analysis using CLC Genomics Workbench: max # of gaps and mismatches 2, minimum average of quality of surrounding bases 15, minimum quality of central base 20, minimum coverage 1, minimum variant frequence 35%. DIP analysis parameters using CLC Genomics Workbench: minimum coverage 4, minimum variant frequency 35%. *De novo* assembly was completed using Newbler (454 Life Sciences Corporation, release 2.3). Default settings were used for *De novo* assembly and reducing the amount of sequences to gain 22X coverage of a ∼50 Kb genome was performed randomly. Following *De novo* assembly, reference assembly (as described above) was performed using all contigs generated to assess genome coverage. Bioinformatic normalization was performed by reducing the redundancy of reads in genomic regions of high coverage via clustering using cd-hit-est [47].The contig N50 (bp) was recorded as an assembly metric and represents the length of the smallest contig in the set that contains the fewest (largest) contigs whose combined length represents at least 50% of the assembly.

## Supporting Information

Table S1 **Primers specific for phages T4 and lambda loci used in multiplex PCR to identify phage isolated.**
(PDF)

Table S2 **Statistics following SVG methodology on 16 test samples.** CLSM numbers corresponds to viruses detected during microscopy, MDA refers to a positive (+) or negative (−) when amplification was detected by gel electrophoresis of wells containing viral particles. A positive specific PCR is denoted by the genotype obtained after multiplex PCR of the amplified genomic material.
(PDF)

Table S3 **Reference mapping statistics (all sequence lengths are given in bp).**
(PDF)

Table S4 **BLAST analysis of unmapped read sequences following reference mapping.**
(PDF)

Table S5 **Allele variation analysis using CLC Genomics Workbench.**
(PDF)

Table S6 ***De novo*** **assembly statistics.**
(PDF)

Table S7 **Contaminants found in 16S PCR analysis of MDA reactions.**
(PDF)

## References

[1] Raghunathan A, Ferguson HR, Bornarth CJ, Song W, Driscoll M (2005). Genomic DNA amplification from a single bacterium.. Appl Environ Microbiol.

[2] Lasken RS (2007). Single-cell genomic sequencing using Multiple Displacement Amplification.. Curr Opin Microbiol.

[3] Ishoey T, Woyke T, Stepanauskas R, Novotny M, Lasken RS (2008). Genomic sequencing of single microbial cells from environmental samples.. Curr Opin Microbiol.

[4] Falkowski PG, Fenchel T, Delong EF (2008). The microbial engines that drive Earth's biogeochemical cycles.. Science.

[5] Amon RMW, Benner R (1996). Bacterial utilization of different size classes of dissolved organic matter.. Limnology and Oceanography.

[6] Lindell D, Jaffe JD, Johnson ZI, Church GM, Chisholm SW (2005). Photosynthesis genes in marine viruses yield proteins during host infection.. Nature.

[7] Azam F, Fenchel T, Field JG, Gray JS, Meyer Reil LA (1983). The ecological role of water-column microbes in the sea.. Mar Ecol Prog Ser.

[8] Podar M, Abulencia CB, Walcher M, Hutchison D, Zengler K (2007). Targeted access to the genomes of low-abundance organisms in complex microbial communities.. Appl Environ Microbiol.

[9] Lasken RS, Raghunathan A, Kvist T, Ishoy T, Westermann P, Huges S, Lasken R (2005). Multiple displacement amplification from single bacterial cells.. Whole Genome Amplification: *Methods Express*.

[10] Ishey T, Kvist T, Westermann P, Ahring BK (2006). An improved method for single cell isolation of prokaryotes from meso-, thermo- and hyperthermophilic environments using micromanipulation.. Applied Microbiology and Biotechnology.

[11] Dean FB, Nelson JR, Giesler TL, Lasken RS (2001). Rapid amplification of plasmid and phage DNA using Phi 29 DNA polymerase and multiply-primed rolling circle amplification.. Genome Res.

[12] Dean FB, Hosono S, Fang L, Wu X, Faruqi AF (2002). Comprehensive human genome amplification using multiple displacement amplification.. Proc Natl Acad Sci U S A.

[13] Hosono S, Faruqi AF, Dean FB, Du Y, Sun Z (2003). Unbiased whole-genome amplification directly from clinical samples.. Genome Res.

[14] Zhang K, Martiny AC, Reppas NB, Barry KW, Malek J (2006). Sequencing genomes from single cells by polymerase cloning.. Nat Biotechnol.

[15] Marcy Y, Ishoey T, Lasken RS, Stockwell TB, Walenz BP (2007). Nanoliter reactors improve multiple displacement amplification of genomes from single cells.. PLoS Genet.

[16] Rodrigue S, Malmstrom RR, Berlin AM, Birren BW, Henn MR (2009). Whole genome amplification and de novo assembly of single bacterial cells.. PLoS One.

[17] Woyke T, Xie G, Copeland A, Gonz√°lez JM, Han C (2009). Assembling the Marine Metagenome, One Cell at a Time.. PLoS One.

[18] Hutchison CA, Smith HO, Pfannkoch C, Venter JC (2005). Cell-free cloning using phi 29 DNA polymerase.. Proc Natl Acad Sci U S A.

[19] Edwards RA, Rohwer F (2005). Viral metagenomics.. Nature Reviews Microbiology.

[20] Rohwer F, Prangishvili D, Lindell D (2009). Roles of viruses in the environment.. Environ Microbiol.

[21] Fauci AS (1988). THE HUMAN IMMUNODEFICIENCY VIRUS - INFECTIVITY AND MECHANISMS OF PATHOGENESIS.. Science.

[22] Lower R, Lower J, Kurth R (1996). The viruses in all of us: Characteristics and biological significance of human endogenous retrovirus sequences.. Proc Natl Acad Sci U S A.

[23] Breitbart M, Salamon P, Andresen B, Mahaffy JM, Segall AM (2002). Genomic analysis of uncultured marine viral communities.. Proc Natl Acad Sci U S A.

[24] Breitbart M, Felts B, Kelley S, Mahaffy JM, Nulton J (2004). Diversity and population structure of a near-shore marine-sediment viral community.. Proc Biol Sci.

[25] Angly FE, Felts B, Breitbart M, Salamon P, Edwards RA (2006). The marine viromes of four oceanic regions.. PLoS Biol.

[26] Culley AI, Lang AS, Suttle CA (2006). Metagenomic analysis of coastal RNA virus communities.. Science.

[27] Bench SR, Hanson TE, Williamson KE, Gosh D, Radosovich M (2007). Metagenomic characterization of Chesapeake Bay virioplankton.. Appl Environ Microbiol.

[28] Williamson SJ, Rusch DB, Yooseph S, Halpern AL, Heidelberg KB (2008). The Sorcerer II Global Ocean Sampling Expedition: metagenomic characterization of viruses within aquatic microbial samples.. PLoS One.

[29] Staley JT, Konopka A (1985). Measurement of in situ activities of nonphotosynthetic microorganisms in aquatic and terrestrial habitats.. Annu Rev Microbiol.

[30] Fuhrman JA, Campbell L (1998). Microbial microdiversity.. Nature.

[31] Whitman WB, Coleman DC, Wiebe WJ (1998). Prokaryotes: the unseen majority.. Proc Natl Acad Sci U S A.

[32] Rappe MS, Giovannoni SJ (2003). The uncultured microbial majority.. Annu Rev Microbiol.

[33] Brussaard CP (2004). Optimization of procedures for counting viruses by flow cytometry.. Appl Environ Microbiol.

[34] Marie D, Brussaard CPD, Thyrhaug R, Bratbak G, Vaulot D (1999). Enumeration of marine viruses in culture and natural samples by flow cytometry.. Appl Environ Microbiol.

[35] Brussaard CPD, Marieb D, Bratbak G (2000). Flow cytometric detection of viruses.. J Virol Meth.

[36] Brussaard CP (2009). Enumeration of bacteriophages using flow cytometry.. Methods Mol Biol.

[37] Noble RT, Fuhrman JA (1998). Use of SYBR Green I for rapid epifluorescence counts of marine viruses and bacteria.. Aquat Microb Ecol.

[38] Luef B, Neu TR, Peduzzi P (2009). Imaging and quantifying virus fluorescence signals on aquatic aggregates: a new method and its implication for aquatic microbial ecology.. FEMS Microbiol Ecol.

[39] Tzvetkov MV, Becker C, Kulle B, Nurnberg P, Brockmoller J (2005). Genome-wide single-nucleotide polymorphism arrays demonstrate high fidelity of multiple displacement-based whole-genome amplification.. Electrophoresis.

[40] Pinard R, de Winter A, Sarkis GJ, Gerstein MB, Tartaro KR (2006). Assessment of whole genome amplification-induced bias through high-throughput, massively parallel whole genome sequencing.. BMC Genomics.

[41] Miller JR, Koren S, Sutton G (2010). Assembly algorithms for next-generation sequencing data.. Genomics.

[42] Panelli S, Damiani G, Espen L, Sgaramella V (2005). Ligation overcomes terminal underrepresentation in multiple displacement amplification of linear DNA.. Biotechniques.

[43] Blainey PC, Quake SR Digital MDA for enumeration of total nucleic acid contamination.. Nucleic Acids Research.

[44] Winget DM, Wommack KE (2008). Randomly amplified polymorphic DNA PCR as a tool for assessment of marine viral richness.. Appl Environ Microbiol.

[45] Meng X, Benson K, Chada K, Huff EJ, Schwartz DC (1995). Optical mapping of lambda bacteriophage clones using restriction endonucleases.. Nature Genetics.

[46] Storrie-Lombardi MC, Irwin MJ, von Hippel T, Storrie-Lombardi LJ (1994). Spectral classification with principal component analysis and artificial neural networks.. Vistas in Astronomy.

[47] Li WZ, Godzik A (2006). Cd-hit: a fast program for clustering and comparing large sets of protein or nucleotide sequences.. Bioinformatics.

