# Supplementary material for: Single Virus Genomics: A New Tool for Virus Discovery
Source: PLoS One. 2011 Mar 23;6(3):e17722. doi: 10.1371/journal.pone.0017722 (PMC3059205; doi:10.1371/journal.pone.0017722)
Supplement: Table S1 — Primers specific for phages T4 and lambda loci used in multiplex PCR to identify phage isolated. (PDF) [file pone.0017722.s001.pdf]

Phage	Primer Name	Sequence (5'-3')
lambda	LsieF	TCT GGG TTG GAC TTC TGC TT
lambda	LsieR	TTG GCC TTG GCT TTA TCT CA
lambda	LrepF	GGC TTA TCC CAG GAA TCT GT
lambda	LrepR	TAC CTT CAA CCT CAA GCC AG
lambda	lbdintF	TGA TAC TGT GCC GGA TGA AA
lambda	lbdintR	TTA GGC AGA GAC AGG CGA AT
T4	gp23F	TGG CGC AGT AAC TCA GAT TG
T4	gp23R	CCA TTT CGT TCG GAC CTT TA
