# Supplementary material for: Single Virus Genomics: A New Tool for Virus Discovery
Source: PLoS One. 2011 Mar 23;6(3):e17722. doi: 10.1371/journal.pone.0017722 (PMC3059205; doi:10.1371/journal.pone.0017722)
Supplement: Table S2 — Statistics following SVG methodology on 16 test samples. CLSM numbers corresponds to viruses detected during microscopy, MDA refers to a positive (+) or negative (−) when amplification was detected by gel electrophoresis of wells containing viral particles. A positive specific PCR is denoted by the genotype obtained after multiplex PCR of the amplified genomic material. (PDF) [file pone.0017722.s002.pdf]

Test Slide: T4/lambda						
**Well**	**CLSM**	**MDA**	**PCR**	**CLSM**	**MDA**	**PCR**
1	1	-		0	+	
2	>1	+		>1	+	T4
3	>1	+		1	+	
4	1	+		1	+	
5	>1	+		>1	+	T4
6	0	+		0	+	
7	>1	+		1	+	lambda
8	>1	+	lambda	0	+	
Summary						
**% with ≥1 virus**			75			
**% with positive MDA**			92			
**% with positive specific PCR**			25			
