# Supplementary material for: Single Virus Genomics: A New Tool for Virus Discovery
Source: PLoS One. 2011 Mar 23;6(3):e17722. doi: 10.1371/journal.pone.0017722 (PMC3059205; doi:10.1371/journal.pone.0017722)
Supplement: Table S3 — Reference mapping statistics (all sequence lengths are given in bp). (PDF) [file pone.0017722.s003.pdf]

Total reference length	48,502
GC contents in %	49.9
Total read count	61,406
Mean read length	361.6
Total read length	22,202,436
Total consensus length	48,497
Zero coverage length	5
Minimum coverage	0
Maximum coverage	2,000
Average coverage	436.9
