# Supplementary material for: Single Virus Genomics: A New Tool for Virus Discovery
Source: PLoS One. 2011 Mar 23;6(3):e17722. doi: 10.1371/journal.pone.0017722 (PMC3059205; doi:10.1371/journal.pone.0017722)
Supplement: Table S4 — BLAST analysis of unmapped read sequences following reference mapping. (PDF) [file pone.0017722.s004.pdf]

Taxa	#Hits/Taxa	% Total reads
Phage	552	0.55
Unclassified	16,095	16.11
Pseudomonas	12,971	12.98
Escherichia	2,762	2.76
Shigella	811	0.82
Xanthomonas	419	0.42
Ralstonia	364	0.36
Homo sapiens	308	0.31
Rhodobacter	272	0.27
Stenotrophomonas	266	0.26
Aeromonas	238	0.24
Burkholderia	231	0.23
Roseobacter	196	0.20
Populus	117	0.12
Xylella	110	0.11
Flavobacterium	102	0.10
Other Genera	1,755	1.76
