# Supplementary material for: Single Virus Genomics: A New Tool for Virus Discovery
Source: PLoS One. 2011 Mar 23;6(3):e17722. doi: 10.1371/journal.pone.0017722 (PMC3059205; doi:10.1371/journal.pone.0017722)
Supplement: Table S5 — Allele variation analysis using CLC Genomics Workbench. (PDF) [file pone.0017722.s005.pdf]

Reference position	Consensus position	Variation type	Reference	Allele variation	Allele variation frequency	# of Allele variations	Depth Coverage	Locus	Gene Product name	Amino Acid Change	Amino Acid polarity and charge change
138	133	SNP	G	A	100	1	1	--	--	--	--
542	536	SNP	C	T	98.4	127	129	nu1	DNA packaging protein	--	--
20661	20656	SNP	A	G	97.9	142	145	orf206b	hypothetical protein lambdap90	Phe36Ser; Lys338Glu	nonpolar, hydrophobic:polar, uncharged; polar basic:polar acidic
22375	22370	SNP	A	G	83.3	20	24	orf-194		Ser135Gly	--
22444	22439	SNP	A	G	100	167	167	orf-194	putative fiber assembly protein	Lys158Glu	polar basic:polar acidic
23773	23768	SNP	T	G	66.7	2	3	ea47	ea47	Gln49Pro	polar, uncharged:nonpolar hydrophobic
25662	25657	SNP	T	C	100	347	347	ea59	ea59	Met438Val	--
30862	30857	SNP	T	T/C	64.3/35.7	18/10	28	xis	Excisionase	Ser55Gly	--
31016	31011	SNP	T	C	99.5	390	392	xis	Excisionase	Asn61Asp	polar, uncharged:polar acidic
31966	31961	SNP	A	T	99.5	192	193	xis	Excisionase	Asp21Glu	--
34934	34929	SNP	A	G	99.5	188	189	xis	Excisionase	--	--
37870	37865	SNP	T	C/T	50.0/50.0	1/1	2	cl	repressor	Glu24Gly	polar acidic:polar uncharged
45618	45613	SNP	T	C	100	346	346	R	endolysin	--	--
46430	46425	SNP	T	C	99.7	337	338	--	--	--	--
46433	46428	SNP	C	A	100	320	320	--	--	--	--
46464	46459	SNP	G	T	100	180	180	bor	bor protein precursor	Gln97Lys	polar, uncharged:polar basic
46588	46583	SNP	G	A	99.1	210	212	bor	bor protein precursor	--	--
138	133	DIP	G	-	97.3	36	37		--	--	--
14267	14261	DIP	-	G	98.7	232	235		--	--	--
37619	37614	DIP	A	A/-	53.4/37.5	47/33	88	cl	repressor	frameshift	--
39143	39138	DIP	A	A/-	60.5/38.6	69/44	114	O	DNA replication protein	frameshift	--
