# Supplementary material for: Single Virus Genomics: A New Tool for Virus Discovery
Source: PLoS One. 2011 Mar 23;6(3):e17722. doi: 10.1371/journal.pone.0017722 (PMC3059205; doi:10.1371/journal.pone.0017722)
Supplement: Table S6 — De novo assembly statistics. (PDF) [file pone.0017722.s006.pdf]

Assembly	Aligned Reads	Reads Assembled	Singletons	Total Contigs	Largest Contigs (bp)	N50 Contig Size (bp)
22X coverage	2,513	1,910	711	47	15,799	5,324
All reads	91,344	70,034	3,093	1,129	18,286	1,324
cd-hit filtered	41,327	32,139	1,093	109	5,668	1,433
