# Supplementary material for: Single Virus Genomics: A New Tool for Virus Discovery
Source: PLoS One. 2011 Mar 23;6(3):e17722. doi: 10.1371/journal.pone.0017722 (PMC3059205; doi:10.1371/journal.pone.0017722)
Supplement: Table S7 — Contaminants found in 16S PCR analysis of MDA reactions. (PDF) [file pone.0017722.s007.pdf]

Taxa	#Hits/Taxa	% Contaminants
Burkholderia	19	38.0
Stenotrophomonas	8	16.0
Pseudomonas	6	12.0
Achromobacter	4	8.0
Enteric Bacteria cluster	3	6.0
Sediminibacterium	2	4.0
Prevotella	1	2.0
Brochothrix	1	2.0
Shewanella	1	2.0
Ralstonia	1	2.0
Uncultured	1	2.0
Cyanobacteria	1	2.0
SAR11 cluster	1	2.0
Environmental sample	1	2.0
